# Depressive symptoms are associated with oxidative stress in middle-aged women: a cross-sectional study

**DOI:** 10.1186/s13030-016-0066-4

**Published:** 2016-04-26

**Authors:** Asuka Hirose, Masakazu Terauchi, Mihoko Akiyoshi, Yoko Owa, Kiyoko Kato, Toshiro Kubota

**Affiliations:** Department of Obstetrics and Gynecology, Tokyo Medical and Dental University, Yushima 1-5-45, Bunkyo, Tokyo, 113-8510 Japan; Department of Women’s Health, Tokyo Medical and Dental University, Yushima 1-5-45, Bunkyo, Tokyo, 113-8510 Japan

**Keywords:** 8-OHdG, Mood disorder, Menopausal symptoms, Depression, Menopause

## Abstract

**Background:**

Oxidative stress is known to be a factor in various diseases. In this study, we investigated whether physical and psychological symptoms of menopause, cardiovascular parameters, body composition, and lifestyle factors are associated with oxidative stress in middle-aged women.

**Methods:**

This cross-sectional study used baseline data collected in a previous study that examined the effects of a dietary supplement on a variety of health parameters in 95 women aged 40 to 60 years. Participants had been assessed for age, menopausal status, body composition, cardiovascular parameters, physical and psychological symptoms of menopause, and lifestyle factors. Urinary 8-hydroxy-2′-deoxyguanosine (8-OHdG) level, an oxidative stress marker, had also been measured. Dichotomizing 8-OHdG levels as low (≤25 ng/mg creatinine) and high (>25 ng/mg creatinine), we sought to identify the health parameters that are associated with high 8-OHdG level.

**Results:**

Women with a high 8-OHdG level had lower body weight, lower body mass index, lower body fat mass, higher body temperature, scored higher for both anxiety and depression on the Hospital Anxiety and Depression Scale (HADS), and consumed more alcohol. Multiple logistic regression analysis revealed that the HADS-depression subscale (HADS-D) score was the sole independent contributor to high 8-OHdG level (adjusted odds ratio, 1.23 per point increase in HADS-D score; 95 % confidence interval, 1.06–1.45).

**Conclusion:**

Depressive symptom score was shown to be independently associated with high 8-OHdG level in middle-aged women, suggesting a link between mood disorder and oxidative stress.

**Trial registration:**

UMIN-CTR UMIN000009353.

## Background

Health promotion for middle-aged women is gaining attention due to the various health care issues raised with the advent of an aging society. Women in this age group are not only bothered by menopausal symptoms, such as hot flashes, night sweats, vaginal dryness, anxiety, depression, and insomnia, but are also at increased risk for cardiovascular disease (CVD), central obesity, hypertension, dyslipidemia, and diabetes [1–4].

Oxygen is vital for all aerobic organisms. During aerobic metabolism, oxygen is reduced into water, and in the process, reactive oxygen species (ROS), such as superoxide anion, hydrogen peroxide, hydroxyl radical, and singlet oxygen, are generated [5]. ROS are highly reactive molecules that chemically change the structures of proteins, lipids, and nucleic acids, and cause cell and tissue damage [6, 7]. Organisms have an antioxidant reaction to protect themselves against ROS through detoxification mechanisms mediated by antioxidants and antioxidant enzymes [5]. Excessive ROS production or antioxidant deficiency leads to damage of aerobic organisms; imbalance between oxidants and antioxidants in favor of the former is referred to as oxidative stress [8, 9].

First introduced by Sies in 1985 [10], oxidative stress has been found to contribute to the development of various diseases, including cancer [11], atherosclerosis [12, 13], myocardial infarction [13], Parkinson’s disease [14], Alzheimer’s disease [15], inflammatory airway diseases [16], and age-related illness [17]. The severity of oxidative stress can be quantified by measuring the concentration of specific biomarkers, one of which is 8-hydroxy-2′-deoxyguanosine (8-OHdG), an index of oxidatively damaged DNA [18, 19]. Recently, a novel automatic analyzer for competitive immunochromatography was developed, making measurement of urinary 8-OHdG level much quicker and easier than before.

The aim of this study was to investigate whether physical and psychological symptoms of menopause, cardiovascular parameters, body composition, and lifestyle factors are associated with oxidative stress in middle-aged women using urinary 8-OHdG level as a marker.

## Methods

### Study population

We performed a cross-sectional analysis using the baseline data of a previous study [20] conducted at the Menopause Clinic of Tokyo Medical and Dental University to examine the effects of a dietary supplement on a variety of health parameters in 95 Japanese women aged 40 to 60 years. The participants were recruited through advertisements posted in our hospital and the patients’ own social network. Women who scored positive for at least one symptom item on the Menopausal Health-Related Quality of Life (MHR-QOL) Questionnaire were eligible for the study, and those who were receiving menopausal hormone therapy were excluded. Collected data included age, menopausal status, lifestyle factors, physical and psychological symptoms of menopause, body composition, cardiovascular parameters, and urinary 8-OHdG level. The study protocol was reviewed and approved by the Medical Research Ethics Committee of Tokyo Medical and Dental University (No. 1359), and written informed consent was obtained from all participants. The study was conducted in accordance with the Declaration of Helsinki.

Regarding menopausal status, participants were classified as follows: premenopausal (regular menstrual cycles in the past 3 months), perimenopausal (a menstrual period within the past 12 months but a missed period or irregular cycles in the past 3 months), postmenopausal (no menstrual period in the past 12 months), or surgically induced menopause (hysterectomy).

### Body composition and cardiovascular parameters

Body composition, including height, weight, body mass index, fat mass, and muscle mass, was assessed using a body composition analyzer (MC190-EM; Tanita, Tokyo, Japan). Cardiovascular parameters, including systolic and diastolic blood pressure, heart rate, and arterial stiffness index (cardio-ankle vascular index), were measured using a vascular screening system (VS-1000; Fukuda Denshi Co., Tokyo, Japan).

### Menopausal symptoms

Menopausal symptoms were evaluated using the MHR-QOL Questionnaire, Hospital Anxiety and Depression Scale (HADS), and Athens Insomnia Scale (AIS). The MHR-QOL Questionnaire, developed and validated in our clinic, is a modification of the Women’s Health Questionnaire [21, 22] that contains 38 items scored on a four-point or binary scale covering four major domains (physical health, mental health, life satisfaction, and social involvement) of a woman’s health during menopause. The physical health domain consists of nine items that assess somatic (sickness/nausea, dizzy spells, tingling/numbness, backache/pain in limbs, tiredness, and headaches), urinary (urinary frequency), and vasomotor (hot flashes and night sweats) symptoms. The mental health domain consists of 12 items that assess symptoms of loss of interest in things, lack of enjoyment, low energy, depressed mood, poor memory, difficulty in concentration, frightened/panicky feelings, feeling tense/wound up, dissatisfaction with sexual relationship, difficulty in initiating sleep, nonrestorative sleep, and low self-esteem. Higher scores on the items indicate worse physical function (0 points, none to once per month; 1 point, 1–2 times per week; 2 points, 3–4 times per week; 3 points, almost every day). The total scores of the physical and mental health domains were designated the “physical symptom score” and “psychological symptom score” respectively. The HADS, developed by Zigmond and Snaith [23], is a reliable instrument for screening clinically significant anxiety and depressive symptoms in women visiting a general medicine clinic; it has been translated into Japanese by Kitamura et al. [24]. The AIS was developed as a brief and easy-to-administer self-assessment questionnaire for determining the severity of insomnia defined according to the International Classification of Diseases 10th Revision. The internal consistency and test-retest reliability of the AIS have been confirmed previously [25]. Detailed information about the MHR-QOL Questionnaire, HADS, and AIS is provided elsewhere [20].

### Oxidative stress

Urinary 8-OHdG level was measured by competitive immunochromatography using a novel automatic analyzer (ICR-001; Techno Medica, Tokyo, Japan). The participants produced urine samples at our clinic, which were instantly centrifuged at 3000 rpm for 5 min. After dilution twice with distilled water, 8-OHdG (ng) and creatinine (mg) concentration were measured to calculate 8-OHdG level corrected by creatinine (ng/mg creatinine). In the present study, we dichotomized 8-OHdG levels as low (≤25 ng/mg creatinine) and high (>25 ng/mg creatinine) according to previous research that analyzed 140 samples collected from healthy workers at a coke oven plant [26]. In that study, the mean ± SD value measured by using enzyme-linked immunosorbent assay (ELISA) was 14.9 ± 9.3 ng/mg creatinine in subjects who were employed in the coke operation area and 14.7 ± 12.8 ng/mg creatinine in subjects who worked only in an administrative area. Based on the mean + 1SD value in that study, we arbitrarily set our cut-off index at 25 ng/mg creatinine.

### Statistical analyses

First, all variables were compared between the two 8-OHdG level groups using univariate analysis (unpaired *t* test, chi-square test, and Mann–Whitney *U* test). Then, variables emerging with possible prognostic value for high 8-OHdG level (*P* < 0.20) were entered into stepwise multiple logistic regression analysis. Variables that remained significant (*P* < 0.05) were retained in the final multivariable model and were considered to be associated with oxidative stress in middle-aged women. All statistical analyses were performed using SAS version 9.2 (SAS Institute, Cary, NC, USA).

## Results

The 95 participants were divided into two groups according to urinary 8-OHdG level: 59 women had a low level (≤25 ng/mg creatinine) and 36 had a high level (>25 ng/mg creatinine).

The background characteristics of the women are shown by group in Table 1. First, we examined differences between groups using univariate analysis to select variables with possible prognostic value for high 8-OHdG level. Mean age and menopausal status were similar between groups. Women with a high 8-OHdG level tended to have lower body weight (51.0 ± 8.3 kg vs. 53.5 ± 6.5 kg, *P* = 0.111), lower body mass index (20.6 ± 3.4 kg/m^2^ vs. 21.9 ± 2.5 kg/m^2^, *P* = 0.048), lower body fat mass (12.9 ± 6.3 kg vs. 15.1 ± 4.8 kg, *P* = 0.062), higher body temperature (36.4 ± 0.4 °C vs. 36.2 ± 0.5 °C, *P* = 0.123), scored higher for anxiety (6.0 ± 3.4 vs. 4.9 ± 2.6, *P* = 0.100) and depression (5.3 ± 3.6 vs. 3.5 ± 2.3, *P* = 0.018) on the HADS, and scored higher on the MHR-QOL psychological symptom score (8.2 ± 8.9 vs. 4.6 ± 4.7, *P* = 0.054). They also consumed more alcohol (proportion of women who drank daily, 22.2 % vs. 11.9 %, *P* = 0.196) (Table 1).

**Table 1:** Background characteristics of the participants

	Low 8-OHdG level	High 8-OHdG level	*P* value
	(*n* = 59)	(*n* = 36)	
Age, y	49.5 ± 5.1	50.0 ± 5.0	0.637^a^
Menopausal status, %			
Premenopausal	42.4	38.9	0.771^b^
Perimenopausal	22.0	22.2	
Postmenopausal	25.4	33.3	
Surgically induced menopause	10.2	5.6	
Body composition			
Height, cm	156.4 ± 5.3	157.2 ± 4.6	0.434^a^
Weight, kg	53.5 ± 6.5	51.0 ± 8.3	0.111^a^
Body mass index, kg/cm^2^	21.9 ± 2.5	20.6 ± 3.4	0.048^a^
Body fat mass, kg	15.1 ± 4.8	12.9 ± 6.3	0.062^a^
Muscle mass, kg	36.2 ± 2.5	35.9 ± 2.7	0.630^a^
Waist:hip ratio, %	85.6 ± 6.9	85.1 ± 8.4	0.760^a^
Body temperature, °C	36.2 ± 0.5	36.4 ± 0.4	0.123^a^
Cardiovascular parameters			
Systolic blood pressure, mmHg	122.5 ± 14.6	119.4 ± 15.5	0.317^a^
Diastolic blood pressure, mmHg	78.6 ± 10.3	76.9 ± 10.3	0.433^a^
Heart rate, beats/min	63.3 ± 7.5	64.9 ± 9.8	0.382^a^
CAVI	7.16 ± 0.68	7.29 ± 0.83	0.405^a^
MHR-QOL physical symptom score	5.58 ± 3.41	5.81 ± 3.85	0.865^c^
MHR-QOL psychological symptom score	4.63 ± 4.71	8.17 ± 8.85	0.054^c^
HADS-anxiety subscale score	4.86 ± 2.62	6.03 ± 3.36	0.100^c^
HADS-depression subscale score	3.48 ± 2.30	5.25 ± 3.63	0.018^c^
AIS score	4.09 ± 2.99	3.75 ± 3.11	0.524^c^
Lifestyle factors, %			
Working	89.8	83.3	0.362^c^
Exercising regularly	45.8	47.2	1.000^c^
Smoking	8.5	16.7	0.322^c^
Drinking alcohol (daily/by chance/not at all)	11.9/57.6/30.5	22.2/61.1/16.7	0.196^c^

Values are presented as mean ± SD unless otherwise indicated

*AIS* Athens insomnia scale, *CAVI* cardio-ankle vascular index, *HADS* hospital anxiety and depression scale, *MHR-QOL* menopausal health-related quality of life

^a^According to the unpaired *t* test, ^b^According to the chi-square test, ^c^According to the Mann–Whitney *U* test

Next, these variables were entered into multivariate analysis to identify the factors that contribute independently to high 8-OHdG level, except for MHR-QOL psychological symptom scores, as they were represented by HADS-anxiety and depression scores. Stepwise multiple logistic regression analysis revealed that, among these factors, HADS-depression subscale (HADS-D) score was the sole independent contributor to high 8-OHdG level (adjusted odds ratio, 1.23 per point increase in HADS-D score; 95 % confidence interval, 1.06–1.45) (Table 2). Considering the multicollinearity of the three body composition variables, we analyzed the data putting each of them into multiple logistic regression analysis, only to find HADS-depression score was still the sole independent contributor to high 8-OHdG level (data not shown).

**Table 2:** Factors associated with high urinary 8-OHdG level (>25 ng/mg creatinine) according to multiple logistic regression analysis

	Crude OR (95 % CI)	*P* value	Adjusted OR (95 % CI)	*P* value
Weight	0.95 (0.89–1.01)	0.110		
Body mass index	0.84 (0.70–0.98)	0.038		
Body fat mass	0.92 (0.84–1.00)	0.056		
Body temperature	2.16 (0.84–6.13)	0.126		
HADS-anxiety subscale score	1.14 (0.99–1.32)	0.083		
HADS-depression subscale score	1.23 (1.06–1.45)	0.011	1.23 (1.06–1.45)	0.011
Daily alcohol consumption	2.20 (0.72–6.72)	0.166		

*CI* confidence interval, *HADS* hospital anxiety and depression scale, *OR* odds ratio

## Discussion

In this cross-sectional analysis of 95 middle-aged women, we investigated the associations between various health parameters and oxidative stress and revealed that the depressive symptom score was independently associated with high urinary 8-OHdG level, suggesting a link between mood disorder and oxidative stress. The result that all the variables except depressive symptom score did not remain in the final model suggests that their association with oxidative stress was cofounded with depressive symptoms.

In order to evaluate the state of oxidative stress, various biomarkers have been developed to assess the damage induced by ROS or the defense provided by antioxidants. Well-known biomarkers include malondialdehyde, hexanoyl-lysine, isoprostanes, and protein carbonyls, which are produced through oxidative damage of lipids and proteins, while antioxidant enzymes include superoxide dismutase, catalase, glutathione peroxidase, and glutathione [8, 27].

8-OHdG, deoxyguanosine hydroxylated at the C-8 position, is a biomarker of oxidative DNA damage [18, 19]. First reported by Kasai et al. in 1984 [28], it has become one of the most widely used markers of oxidative stress. Chemical carcinogens, tumor-promoting agents, and lifestyle factors, such as smoking and drinking, have been reported to increase 8-OHdG level in human organ and leukocyte DNA [18, 19, 29]. Urinary 8-OHdG level can be measured by cumbersome methods, such as high-performance liquid chromatography with electrochemical detection, gas chromatography with mass spectrometry, liquid chromatography with tandem mass spectrometry, and ELISA [30]. A recently developed automatic analyzer for competitive immunochromatography, ICR-001, has made measurement of urinary 8-OHdG level much quicker and easier than before. Thus far, three research papers have been published using the ICR-001 system to measure urinary 8-OHdG levels [31–33].

For middle-aged women, depression is an important health care issue. Although, the main predictor of depression during the perimenopausal period is the symptom before menopause in addition to other psychosocial factors, several studies have suggested that new-onset depressive symptoms increase during the transition to menopause [34]. One study also revealed that fluctuations rather than absolute levels in estradiol and follicle-stimulating hormone in postmenopausal women are associated with increased risk of depressive symptoms [35]. A meta-analysis of 23 studies was recently performed to evaluate the association between depression and oxidative stress and/or antioxidant status in general [36]. Depressive symptoms were found to be associated with increased oxidative stress and lower antioxidant status; however, the measures of oxidative stress and antioxidant status, the assessment of depressive symptoms, and the sample populations were heterogeneous. Some studies using peripheral leukocytes [37], sera [6, 38, 39] or urine samples [40, 41] have revealed an association between depressive symptoms and high 8-OHdG level. Regarding the two studies using urine samples, one investigated the association between 8-OHdG level with major depression and myalgic encephalomyelitis/chronic fatigue syndrome [40], while the other assessed the urinary excretion of the oxidative stress marker throughout the menstrual cycle in young women [41]. The present study was the first to use a novel automatic analyzer of urinary 8-OHdG level to investigate the association between depressive symptoms and oxidative stress in middle-aged women.

Although the mechanisms linking depressive symptoms to oxidative stress remain unknown, some possibilities are suggested. First, depressed women may have lifestyles or behaviors that increase oxidative stress. Second, depressive symptoms may lead to increased production of ROS, which would contribute to oxidative stress. Third, depressive symptoms may weaken the antioxidant defense system. Fourth, the fluctuations in estradiol and follicle-stimulating hormone, which are suggested to be associated with depressive symptoms [35], may also increase oxidative stress. Finally, high oxidative stress may lower serotonin and norepinephrine levels, which would lead to depressive symptoms. In the present study, we negated the possibility that other factors such as age, menopausal status, lifestyle factors (including working, exercise, smoking, and alcohol consumption), physical and psychological symptoms of menopause, body composition, and cardiovascular parameters were confounding. Depressive symptoms and oxidative stress may affect bidirectionally; thus, further research is required to clarify the underlying mechanisms that evaluates several oxidative stress biomarkers, including resultant oxidative products and antioxidant enzymes simultaneously, as well as estradiol, follicle-stimulating hormone, serotonin, and norepinephrine levels.

The present study has some limitations: (1) the number of participants was relatively small, and (2) all participants were relatively healthy women. In order to determine the association between oxidative stress and depressive symptoms, further studies with a larger sample including middle-aged women with more severe depressive symptoms are warranted.

## Conclusion

Depressive symptom score was shown to be independently associated with high 8-OHdG level in middle-aged women, suggesting a link between mood disorder and oxidative stress in this population.

## Ethics approval and consent to participate

The study protocol was reviewed and approved by the Medical Research Ethics Committee of Tokyo Medical and Dental University (No. 1359), and written informed consent was obtained from all participants. The study was conducted in accordance with the Declaration of Helsinki.

## Consent for publication

Not applicable.

## Availability of data and materials

The datasets are available upon reasonable request.

## Abbreviations

AIS: Athens insomnia scale
CVD: cardiovascular disease
ELISA: enzyme-linked immunosorbent assay
HADS: hospital anxiety and depression scale
MHR-QOL: menopausal health-related quality of life
ROS: reactive oxygen species
8-OHdG: 8-hydroxy-2′-deoxyguanosine
